# A mutational hotspot in *TUBB2A* associated with impaired heterodimer formation and severe brain developmental disorders

**DOI:** 10.3389/fncel.2025.1664953

**Published:** 2025-09-25

**Authors:** Gabriele Di Pasquale, Jacopo Colella, Carola P. Di Cataldo, Miguel A. Soler, Sara Fortuna, Emma Mizrahi-Powell, Mathilde Nizon, Benjamin Cognè, Valentina Turchetti, Giuseppe D. Mangano, Francesco F. Comisi, Corrado Cecchetti, Alessandra Giliberti, Rosaria Nardello, Piero Pavone, Raffaele Falsaperla, Gabriella Di Rosa, Gilad D. Evrony, Maurizio Delvecchio, Mariasavina Severino, Andrea Accogli, Alessandro Vittori, Vincenzo Salpietro

**Affiliations:** 1 Department of Biotechnological and Applied Clinical Sciences, Academic Unit of Pediatrics, University of L'Aquila, L'Aquila, Italy; 2 Department of Mathematics, Computer Science and Physics, University of Udine, Udine, Italy; 3 Italian Institute of Technology (IIT), Genova, Italy; 4 Center for Human Genetics and Genomics, New York University Grossman School of Medicine, New York, NY, United States; 5 Nantes Université, CHU Nantes, Service de Génétique Médicale, Nantes, France; 6 Nantes Université, CNRS, INSERM, l'Institut du Thorax, Nantes, France; 7 Department of Neuromuscular Disorders, UCL Queen Square Institute of Neurology, London, United Kingdom; 8 Department of Medicine and Surgery, University of Enna Kore, Enna, Italy; 9 Pediatric Clinic and Rare Diseases, Microcitemico Hospital "A. Cao", University of Cagliari, Cagliari, Italy; 10 Department of Anesthesia, Critical Care and Pain Medicine, ARCO, Ospedale Pediatrico Bambino Gesù IRCCS, Rome, Italy; 11 Department of Health Promotion, Mother and Child Care, Internal Medicine and Medical Specialities "G. D'Alessandro", University of Palermo, Palermo, Italy; 12 Department of Pediatrics, University of Catania, Catania, Italy; 13 Department of Pediatrics, University of Ferrara, Ferrara, Italy; 14 Unit of Child Neurology and Psychiatry, Maternal-Infantile Department, University of Messina, Messina, Italy; 15 Department of Pediatrics, Department of Neuroscience and Physiology, Institute for Systems Genetics, Perlmutter Cancer Center and Neuroscience Institute, New York University Grossman School of Medicine, New York, NY, United States; 16 Neuroradiology Unit, IRCCS Istituto Giannina Gaslini, Genoa, Italy; 17 Division of Medical Genetics, Department of Medicine, McGill University Health Center, Montreal, QC, Canada; 18 Department of Human Genetics, McGill University, Montreal, QC, Canada; 19 European Brain Research Institute “Rita Levi-Montalcini” Viale Regina Elena, Rome, Italy

**Keywords:** TUBB2A, neurodevelopmental disorders, tubulinopathies, intellectual disability, behavioral disorders, microcephaly, protein modeling

## Abstract

**Introduction:**

Microtubules are essential components of the neuronal cytoskeleton. The *α*- and *β*-tubulins, variably expressed in the central nervous system, play key roles in neurogenesis and brain development. Pathogenic variants in *TUBB2A* have recently been identified as an ultra-rare cause of pediatric neurodevelopmental disorders (NDDs). However, the neurological and behavioral manifestations, genotype–phenotype correlations, and underlying disease mechanisms remain poorly understood due to the limited number of reported families.

**Methods:**

We describe a cohort of families presenting with microcephaly, global developmental delay, speech impairment, seizures and/or EEG abnormalities, movement disorders and severe behavioral disorders. Clinical assessments and brain imaging studies were conducted over a 10-year follow-up period. Genetic analysis was performed via whole-exome sequencing (WES), and structural modeling was used to investigate the functional impact of the identified variants.

**Results:**

WES revealed a novel recurrent heterozygous pathogenic variant in *TUBB2A* (NM_001069.3:c.1172G > A; NP_001060.1:p.Arg391His), identified as the cause of disease in multiple affected individuals from unrelated families. Comparative analysis with previously reported *TUBB2A de novo* variants confirmed that this novel recurrent mutation affects a highly conserved Arg391 residue within the longitudinal E-site heterodimer interface. Computational modeling demonstrated that the variant disrupts *α*/*β*-tubulin heterodimer formation, impairing binding stability at this critical interaction site.

**Discussion:**

Our findings expand the phenotypic and genotypic spectrum of *TUBB2A*-related disorders and identify Arg391 as a mutational hotspot linked to severe brain developmental disorders due to aberrant tubulin dynamics, highlighting the disruption of the *α*/*β*-tubulin heterodimer formation as the disease mechanism associated to this novel hotspot variant. These results provide new insights into disease mechanisms and offer a foundation for potential future therapeutic approaches aimed at stabilizing *α*/*β*-tubulin interactions.

## 1 Introduction

Microtubules are critical structural and functional components of the neuronal cytoskeleton, orchestrating a wide array of processes essential for brain development. These dynamic polymers are composed of α- and β-tubulin heterodimers, which assemble into highly organized arrays that support neurogenesis, neuronal migration, differentiation, and the formation of synaptic architecture and connectivity (Janke and Magiera, 2020; Breuss et al., 2017). The functional diversity of microtubules is further modulated by the differential expression of tubulin isotypes and a range of post-translational modifications, which together fine-tune neuronal migration and intracellular dynamics, leading to the correct development of the brain (Moutin et al., 2021; Wloga et al., 2017; Tantry and Santhakumar, 2023). Proper microtubule function underpins essential developmental processes, including the proliferation of neural progenitors, guidance of immature neurons from periventricular zones to the cortical plate, stabilization of axonal projections, and the formation of synaptic networks (Tantry and Santhakumar, 2023; Kumar et al., 2010; Maillard et al., 2023). To date, at least ten *α*-tubulin isotypes (i.e., *TUBA1A*, *TUBA1B*, *TUBA1C*, *TUBA4A*, *TUBA4B*, *TUBA3C*, *TUBA3D*, *TUBA3E*, *TUBA8*, and *TUBAL3*) and seven *β*-tubulin isotypes (i.e., *TUBB*, *TUBB2A*, *TUBB2B*, *TUBB3*, *TUBB4A*, *TUBB4B*, and *TUBB6*) have been identified (Gonçalves et al., 2018; Park et al., 2021). Their interactions with key microtubule-associated proteins—including DCX, LIS1, DYNC1H1, FLNA, and Tau—are indispensable for cytoskeletal integrity and neuronal polarity.

Neurodevelopmental disorders (NDDs) comprise a heterogeneous group of frequently overlapping conditions, including developmental delay (DD), intellectual disability (ID), speech and motor impairments, behavioral dysregulation, and autism spectrum disorder (ASD) (Salpietro et al., 2019; de Masfrand et al., 2024; Kotchetkov et al., 2023). Despite the advent of next-generation sequencing (NGS), the molecular underpinnings of NDDs remain unresolved in a substantial proportion of patients (Salpietro et al., 2018; Iacomino et al., 2024). A deeper understanding of the genetic architecture and disrupted biological pathways is essential to improve diagnostic precision and inform targeted interventions.

In recent years, pathogenic variants in tubulin-encoding genes have emerged as causative factors in a broad spectrum of neurodevelopmental and neurological disorders. Given their fundamental roles in neuroanatomical organization, tubulin gene mutations have been strongly linked to malformations of cortical development (MCDs), defining a growing group of disorders collectively termed *tubulinopathies* (Severino et al., 2020; Bahi-Buisson et al., 2014; Romaniello et al., 2018). The resultant phenotypes reflect diverse pathogenic mechanisms, including impaired neuroblast proliferation, aberrant neuronal migration, defective axonal pathfinding, and disrupted synaptogenesis (Breuss et al., 2017; Bahi-Buisson et al., 2014; Oegema et al., 2015; Breuss et al., 2017). Clinically, tubulinopathies encompass a broad neurodevelopmental spectrum including microcephaly, variable neurodevelopmental impairment including motor delay, intellectual disability, speech difficulties and broad behavior abnormalities, as well as frequent movement disorders, stereotypies and epilepsy (Brock et al., 2021; Schmidt et al., 2021). Neuroimaging typically reveals characteristic abnormalities, such as polymicrogyria, lissencephaly-pachygyria, dysgyria, agenesis of the corpus callosum, basal ganglia dysmorphism, brainstem asymmetry, and cerebellar hypoplasia or dysplasia (Breuss et al., 2017; Patel et al., 2017; Cushion et al., 2014; Ejaz et al., 2017).

While variants in *TUBA1A*, *TUBB2B*, and *TUBB3* account for the majority (>90%) of known tubulinopathy-associated mutations (Gonçalves et al., 2018; Bahi-Buisson et al., 2014), Pathogenic variants in other tubulin genes (including *TUBB2A*) are considerably rarer and less well characterized. Although initial reports suggested stereotyped phenotypes for mutations in specific tubulin genes, subsequent studies have challenged this notion, revealing marked inter- and intra-genic variability (Bahi-Buisson et al., 2014; Amrom et al., 2014; Zanni et al., 2013; Guerrini et al., 2012; Cushion et al., 2013).

*TUBB2A* encodes a *β*-tubulin isotype predominantly expressed in the developing brain, although its relative expression appears lower compared to other isoforms (Breuss et al., 2012). Pathogenic variants in *TUBB2A* are extremely rare and currently account for only a small fraction of tubulinopathy cases (Schmidt et al., 2021). To date, only 24 individuals have been reported with *TUBB2A*-related disease, showing wide variability in both neurodevelopmental trajectories and neuroimaging findings, ranging from near-normal cortical architecture to severe cortical malformations (Cushion et al., 2014; Ejaz et al., 2017; Brock et al., 2021; Schmidt et al., 2021; Cai et al., 2020; Sferra et al., 2018; Rodan et al., 2017; Lee et al., 2014).

Here, we expand the phenotypic and mutational spectrum of *TUBB2A*-associated tubulinopathy by presenting four additional individuals harboring a novel heterozygous missense variant (NM_001069.3:c.1172G > A; NP_001060.1:p.Arg391His). This variant affects a highly conserved residue in the C-terminal domain of *β*-tubulin and highlights a mutational hotspot with significant implications for microtubule dynamics and *α*/β-tubulin heterodimer formation, leading to disorders in brain development and function.

## 2 Materials and methods

### 2.1 Patient recruitment and clinical evaluation

Four individuals (from three unrelated families) harboring the same novel *TUBB2A* heterozygous missense variant (NM_001069.3:c.1172G > A; NP_001060.1:p.Arg391His) were enrolled in this study. Two were siblings (Individuals 1 and 2), followed longitudinally at an Italian tertiary care center, and two were unrelated patients (Individuals 3 and 4) identified via international collaborative networks among clinicians, clinical geneticists, researchers and neuroradiologists. Individual 4 was enrolled into the Pediatric Undiagnosed Diseases Program (UDP) at the NYU Grossman School of Medicine. Detailed medical histories, physical and neurological examinations, electroencephalograms (EEGs), and brain magnetic resonance imaging (MRI) were collected for all individuals participating in this study. Neurodevelopmental trajectories were assessed through a structured deep phenotyping protocol, including standardized neuropsychiatric evaluation and follow-up spanning multiple years. Peripheral blood samples were collected from affected individuals and their relatives for genetic testing. DNA extraction was performed using standard protocols at each participating center. Whole-exome sequencing (WES) was conducted to identify potential disease-causing variants. Written informed consent was obtained from legal guardians and the study and associated research protocols received approval from the Review Boards and Bioethics Committees at University College London Hospital (project06/N076).

### 2.2 Whole exome sequencing

Genomic DNA was extracted from peripheral blood samples of affected individuals and their parents. Whole exome sequencing (WES) was performed using the 3B-EXOME platform (3billion Inc., Seoul, South Korea) or similar platforms, which targets the protein-coding regions (exons) of the genome, as well as mitochondrial DNA (mtDNA). Sequencing for families 1 and 2 was carried out on the Illumina NovaSeq X platform with an average depth of coverage of ~100×, ensuring that >95% of targeted regions were covered at ≥20×. The exomes were analyzed using a pipeline designed to detect single nucleotide variants (SNVs), small insertions and deletions (INDELs), and copy number variants (CNVs). Family 3 underwent research reanalysis of clinical WES. Variants were annotated, filtered, and classified using ACMG guidelines. Confirmatory testing for candidate variants was performed using Sanger sequencing where applicable.

### 2.3 Literature review

A systematic literature review was conducted to compile all published cases of *TUBB2A*-associated neurodevelopmental disorders. Clinical, genetic, and radiological features from previously reported individuals were extracted and summarized in Table 1, allowing phenotypic comparison with the newly described cases.

**Table 1:** Clinical, EEG and brain MRI features of *TUBB2A*-related neurodevelopmental disorders.

Feature	Cushion-1	Cushion-2	LEE-1	LEE-2	RODAN	EJAZ	SFERRA	CAI-1	CAI-2	BROCK-1	BROCK-2	BROCK-3	BROCK-4
Sex	M	F	Not reported	Not reported	M	M	M	F	F	M	M	F	M
Variant (protein)	p.Asn247Lys	p.Ala248Val	p.Ile345Phe	p.Gln291Pro	p.Ala248Val	p.Arg262His	p.Asp417Asn	p.Pro243Leu	p.Ala248Val	p.Val49Met	p.His396Tyr	p.Val49Gly	p.Asn89Lys
Inheritance	*De novo*	*De novo*	De novo	De novo	De novo	De novo	De novo	De novo	De novo	De novo	De novo	De novo	De novo
Clinical features	Infantile spasms, severe ID	Hypotonia, epilepsy, ID	DD, epilepsy, infantile spasms, microcephaly and plagiocephaly.	DD, epilepsy, ureteropelvic junction obstruction, hydronephrosis, GHD, Silver-Russell syndrome.	GDD, spastic diplegia, behavioral disorder, axial hypotonia, lower limb hypertonia, reflux	Optic nerve hypoplasia, progressive contractures, axial hypotonia appendicular hypertonia	Progressive spastic paraplegia and ataxia, peripheral neuropathy	Moderate GDD; walk delay, nonverbal	Severe GDD, epilepsy and infantile spasm	Moderate MD	MD	MD	MD, dysmorphisms
Brain MRI	Simplified gyral pattern, dysmorphic corpus callosum and basal ganglia, brainstem hypoplasia vermis hypoplasia.	Dysmorphic corpus callosum	Perisylvian polymicrogyria.	Cortical dysplasia	Pachygyria, thinning of corpus callosum, white matter abnormalities	Perisylvian polymicrogyria and abnormal frontal gyration, thin corpus callosum, asymmetric ventricles	Periventricular white matter abnormalities, vermis athrophy, thinning of corpus callosum	White matter myelination delay, enlarged lateral ventricles	Pachygyria, corpus callosum dysplasia	Normal	Dysgyria, dysmorphic corpus callosum	Dysgyria, dysmorphic basal ganglia dysmorphic corpus callosum	Dysgyria, dysmorphic corpus callosum
Microcephaly	No	No	Yes	NA	No	Yes	NA	No	No	No	Yes	Yes	Yes
Epilepsy-presence	Yes	Yes	Yes	Yes	No	Yes	NA	Yes	Yes	Yes	Yes	Yes	Yes
Epilepsy-onset	Infantile	11 months	Infancy	NA	NA	18 months	NA	6 months	5 months	8 months	5 years	5 months	7 months
NDD	GDD	GDD	DD	GDD	GDD	Severe GDD	Mild ID	GDD	Severe GDD	ID	ID	ID	ID
language	Absent	Absent	Delayed and impaired	Delayed and impaired	Absent	Absent	Mild	Limited (simple words only)	Absent	Absent	Absent	Absent	Absent

Feature	BROCK-5	BROCK-6	BROCK-7	BROCK-8	BROCK-9	BROCK-10	BROCK-11	BROCK-12	SCHMIDT-1	SCHMIDT-2	SCHMIDT-3	Our study IND. 1	Our study IND. 2	Our study IND. 3	Our study IND. 4
Sex	F	M	M	F	F	F	M	M	M	F	F	M	M	F	F
Variant (protein)	p.Arg2His	p.Pro357Leu	p.Pro357Leu	p.Ala248Val	p.Ala248Val	p.Glu410Lys	p.Ser230Leu	p.Ala248Val	p.Gly98Arg	p.Gly98Arg	p.Gly98Arg	p.Arg391His	p.Arg391His	p.Arg391His	p.Arg391His
Inheritance	De novo	De novo	De novo	De novo	De novo	De novo	De novo	De novo	De novo	De novo	De novo	Gonadal Mosaicism	Gonadal mosaicism	De novo	De novo
Clinical features	MD	MD	Severe MD	MD	MD	Epilepsy	MD	MD, dysmorphic features	GDD, feeding difficulties, respiratory infections, hypotonia, ADHD, ASD	GDD, ID, ASD, behavioral disorder, hypotonia, non-ambulatory, nonverbal	Hypotonia, epilepsy, ID, sleep disturbance, ADHD, behavioral disorder	Turricephaly, hypotonia, ataxia, ASD, stereotypies, tiptoe walking	Hypotonia, ADHD, cognitive delay, sleep-related seizures, stereotypies	DD, hypotonia, hyperlaxity, increased reflexes, poor receptive language, nonverbal	Severe GDD, epilepsy, hypotonia, tracheostomy, G-tube dependent, neuromuscular scoliosis
Brain MRI	Corpus callosum hypoplasia	Diffuse dysgyria, hypoplastic corpus callosum, basal ganglia anomaly	Dysgyria, hypoplastic corpus callosum, vermis dysplasia	Hypoplastic vermis	Dysmorphic basal ganglia, enlarged horns	Dysgyria	Dysplastic vermis, enlarged horns	Severe dysgyria, thin corpus callosum	Pachygyria; thinning corpus callosum, white matter anomalies, dysmorphic basal ganglia and thalami	At age 3: Pachygyria. At age 6: white matter abnormalities	At age 4: Pachygyria, thinning of corpus callosum; caudate hypoplasia; basal ganglia dysmorphism gray matter abnormalities	Simplified gyral pattern, temporo-basal dysgyria; abnormal corpus callosum	Simplified gyral pattern, temporo-basal dysgyria; abnormal corpus callosum, white matter hypoplasia	Simplified gyral pattern	At 2 years: simplified gyral pattern, abnormal corpus callosum. At 10 years: cerebellar and vermis atrophy, white matter abnormalities
Microcephaly	Yes	Yes	No	NA	NA	No	No	Yes	No	Yes	NA	Yes	Yes	Yes	Yes
Epilepsy-presence	Yes	Yes	Yes	Yes	Yes	No	Yes	Yes	Yes	No	Yes	No	Yes	No	Yes
Epilepsy-onset	10 months	6 month	2 months	6 months	Neonatal	NA	3 years	4 years	8 years	NA	4 years	NA	7 years	NA	Infancy
NDD	ID	ID	ID	ID	ID	ID	ID	ID	GD, ASD, ADHD	ID, ASD, GDD	ID	GDD, ASD	ID, cognitive delay, ASD	Moderate to severe GDD	Severe GDD
Language	Limited	Absent	Absent	Absent	Absent	Delayed and impaired	Delayed and impaired	Delayed and impaired	Severe impairment	Absent	Limited	Absent	Limited	Absent	Absent

NDD, neurodevelopmental disorder; DD, developmental delay; GDD, global developmental delay; MD, motor delay; ID, intellectual disability; ADHD, attention deficit hyperactivity disorder; ASD, autism spectrum disorder; GHD, growth hormone deficiency; NA, not available.

### 2.4 Computational studies

To assess evolutionary conservation of the *TUBB2A* protein and evaluate the potential functional relevance of the p.Arg391His variant, a multiple sequence alignment was performed using Clustal Omega.^1^

To explore the relevance of *TUBB2A* expression to neurodevelopmental and behavioral phenotypes observed in our cohort, we analyzed transcriptomic data from the Human Protein Atlas Brain Atlas.^2^ Normalized RNA expression levels (nTPM) across 13 major human brain regions were reviewed. This dataset provided insights into the spatial distribution of *TUBB2A* expression, supporting its functional relevance in brain regions implicated in the observed clinical features.

### 2.5 Molecular modeling of the Arg391His variant

To model the structural impact of the *TUBB2A* p.Arg391His variant, heterodimer complexes involving TUBB2A and three major *α*-tubulin isotypes (TUBA1A, TUBA1C, and TUBA4A) were constructed using cryo-EM structures from the Protein Data Bank (PDB IDs: 8ixa, 8ixd, and 8ixf) (Diao et al., 2023). The chains corresponding to the α/*β*-tubulin interface and the associated hydrolyzed GTP molecule were extracted to replicate the non-seam binding regions of the microtubule polymer.

The p.Arg391His mutation was introduced using UCSF Chimera, with the most probable rotamer selected to avoid steric clashes (Pettersen et al., 2004; Shapovalov and Dunbrack, 2011). The protonation state of His391 at physiological pH (7.0) was estimated using the PypKa server (Reis et al., 2020; Reis et al., 2024), which predicted a neutral imidazole ring across all heterodimeric models. Molecular dynamics simulations were prepared using the AMBER99SB force field and TIP3P water model. Ligand parameters were generated using Antechamber with BCC-derived GAFF/AMBER topologies (Maier et al., 2015). Systems were solvated in cubic boxes with a 1.5 nm water buffer and prepared using tleap. GROMACS-compatible topologies were obtained through ACPYPE conversion (Sousa da Silva and Vranken, 2012). Energy minimization was carried out in GROMACS v2021 using the steepest descent algorithm under periodic boundary conditions (Abraham et al., 2015). Electrostatics were computed using the Particle Mesh Ewald method until convergence at a maximum force threshold of 1000.0 kJ/mol/nm. Electrostatic binding free energies and electrostatic complementarity were evaluated using bluues_cplx (Soler et al., 2025), which employs Generalized Born radii (Bashford and Case, 2000; Onufriev and Case, 2019) derived from molecular surface integrals generated by Nanoshaper (Decherchi and Rocchia, 2013).

## 3 Results

### 3.1 Identification of a recurrent ***TUBB2A*** missense variant

**Figure 1:**
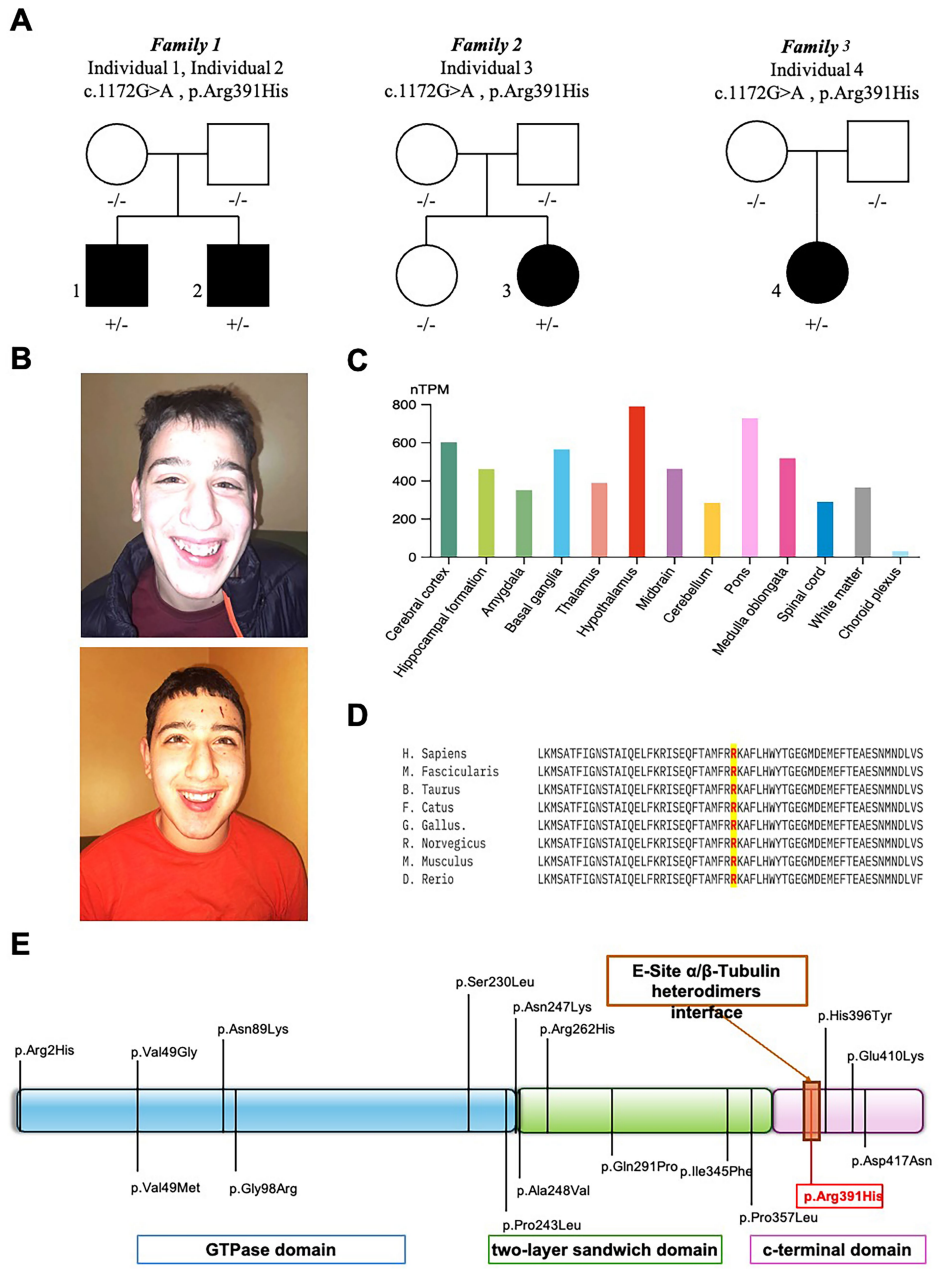
Clinical, molecular, and bioinformatic characterization of *TUBB2A*-related disorder. **(A)** Family pedigrees of affected individuals carrying the heterozygous *TUBB2A* c.1172G > A (p.Arg391His) variant. **(B)** Representative craniofacial features of *TUBB2A*-mutated individuals 1 (upper panel) and 2 (lower panel) showing microcephaly, bitemporal narrowing, and a pointed chin. **(C)** Normalized *TUBB2A* RNA expression levels (nTPM) across 13 human brain regions, based on data from the Human Protein Atlas. Each bar represents the subregion with the highest expression within its region; color coding corresponds to the respective brain region. Notably, the hypothalamus exhibited the highest expression levels. **(D)** Interspecies protein sequence alignment demonstrating complete conservation of the affected residue (Arg391) across vertebrate species, underscoring its evolutionary and functional importance. **(E)** Schematic diagram of the *TUBB2A* protein, illustrating previously reported pathogenic variants in relation to structural domains and highlighting the position of Arg391.

WES of four individuals from three unrelated families (Figure 1) identified a shared heterozygous missense variant in *TUBB2A* (NM_001069.3:c.1172G > A; NP_001060.1:p.Arg391His). In the familial case (Individuals 1 and 2), the variant was absent in both parents, as confirmed by Sanger sequencing, suggesting the possibility of parental germline mosaicism. However, further investigations using additional tissues or sensitive mosaicism detection assays were not conducted, in accordance with the decision of Family 1.

The same heterozygous p.Arg391His variant in *TUBB2A* was also detected in two additional unrelated individuals (Individuals 3 and 4 from Family 2 and 3, Figure 1A) and confirmed *de novo* in trio analyses. The identified hotspot variant p.Arg391His is absent from the gnomAD exome and genome databases^3^ and is predicted to be deleterious by multiple in silico tools, with a high CADD-Phred score of 32. This variant is also highly conserved, as demonstrated by interspecies alignment (Figure 1D) and a GERP++ score of 5.06.

No additional pathogenic or likely pathogenic variants were identified in other known disease-associated and/or NDD-associated genes.

### 3.2 Expression analysis and evolutionary conservation

Transcriptomic data from the Human Protein Atlas revealed *TUBB2A* expression across multiple brain regions, with the highest normalized RNA expression levels (nTPM) observed in the hypothalamus (Figure 1). Notably, *TUBB2A* was expressed in both cortical and subcortical regions, consistent with the widespread neurodevelopmental abnormalities observed in affected individuals. Interspecies protein alignment using Clustal Omega demonstrated that the affected Arg391 residue is fully conserved across vertebrate species, indicating strong evolutionary constraint and potential functional importance.

### 3.3 Structural modeling and functional impact of p.Arg391His

To investigate the structural consequences of the p.Arg391His substitution, we modeled three human *α*/*β*-tubulin heterodimers (TUBA1A–TUBB2A, TUBA1C–TUBB2A, and TUBA4A–TUBB2A) based on cryo-EM structures (PDBs: 8ixa, 8ixd, and 8ixf). In the wild-type state, Arg391 forms a conserved interaction network at the longitudinal E-site interface with *α*-tubulin residues Tyr262, Trp346, and Glu434, stabilizing the heterodimer complex.

**Figure 2:**
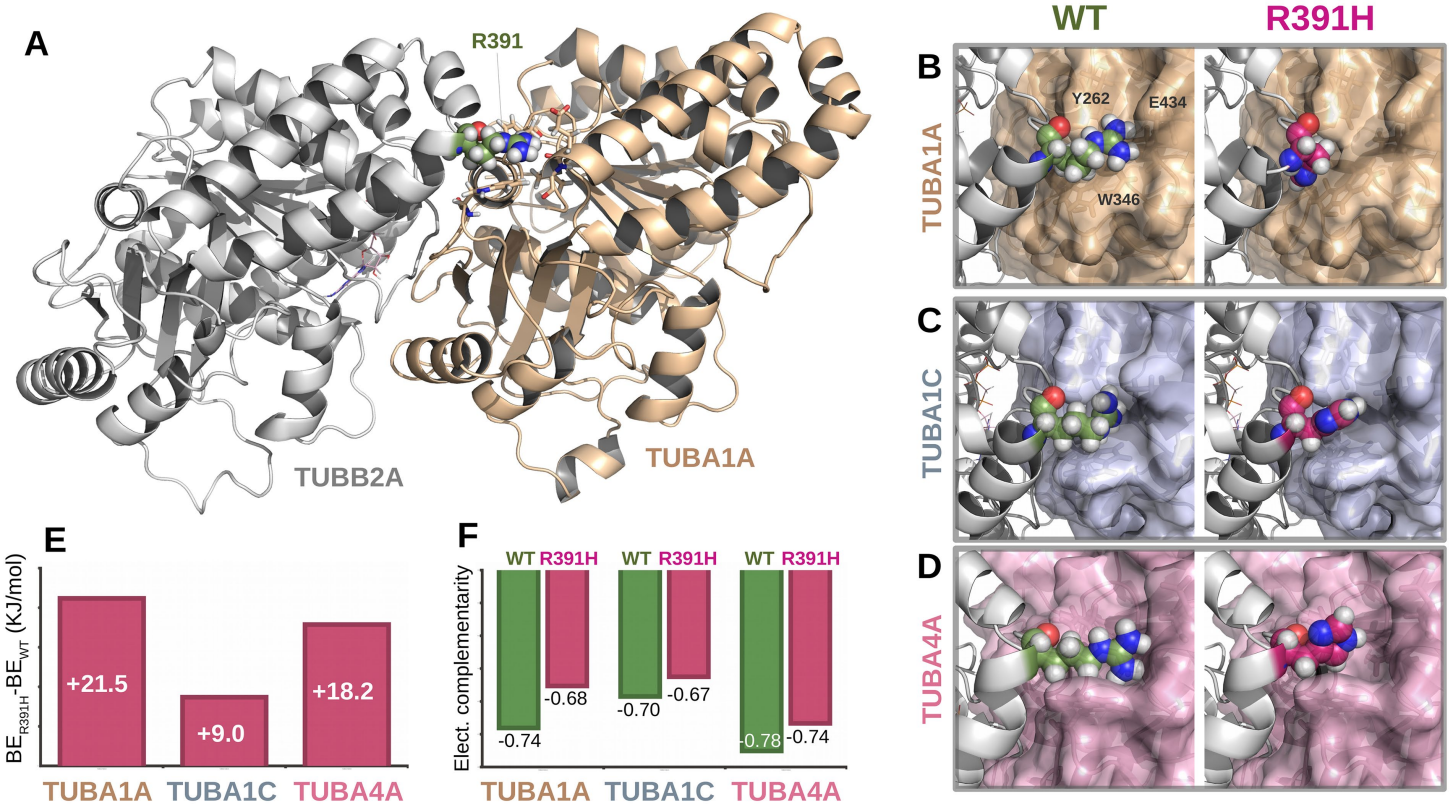
Structural and energetic impact of the *TUBB2A* p.Arg391His variant on α/β-tubulin heterodimerization. **(A)** Structural model of the TUBA1A–TUBB2A heterodimer showing the location of residue Arg391 (green) at the longitudinal E-site interface. **(B–D)** Close-up views of the Arg391 interface in wild-type (left) and mutant p. Arg391His (right) configurations across three α-tubulin isoforms: TUBA1A **(B)**, TUBA1C **(C)**, and TUBA4A **(D)**. In the wild-type complex, Arg391 forms conserved interactions with Tyr262, Trp346, and Glu434 (shown for TUBA1A). These interactions are disrupted upon substitution with histidine. **(E)** Binding energy differences (ΔBE = BE_mut – BE_WT) showing increased free energy in all mutant complexes, indicating reduced binding affinity. **(F)** Electrostatic complementarity at the α/β interface, measured by Pearson correlation coefficients of electrostatic surface potentials. In all cases, mutant dimers (R391H) exhibit lower complementarity compared to their wild-type counterparts.

Introduction of the Arg391His variant disrupted this interface across all three models. Electrostatic calculations of the interfaces revealed increased binding free energies in the mutant dimers compared to wild type, reflecting reduced heterodimer stability (Figure 2). Electrostatic complementarity, assessed via Pearson correlation of electrostatic potential surfaces, was consistently lower in mutant complexes, confirming loss of favorable electrostatic interactions. While minor gains in solvation energy were observed, these did not offset the destabilizing effects of the Arg391His substitution on α/*β*-tubulin dimerization.

### 3.4 Clinical presentations

#### 3.4.1 Individual 1

**Figure 3:**
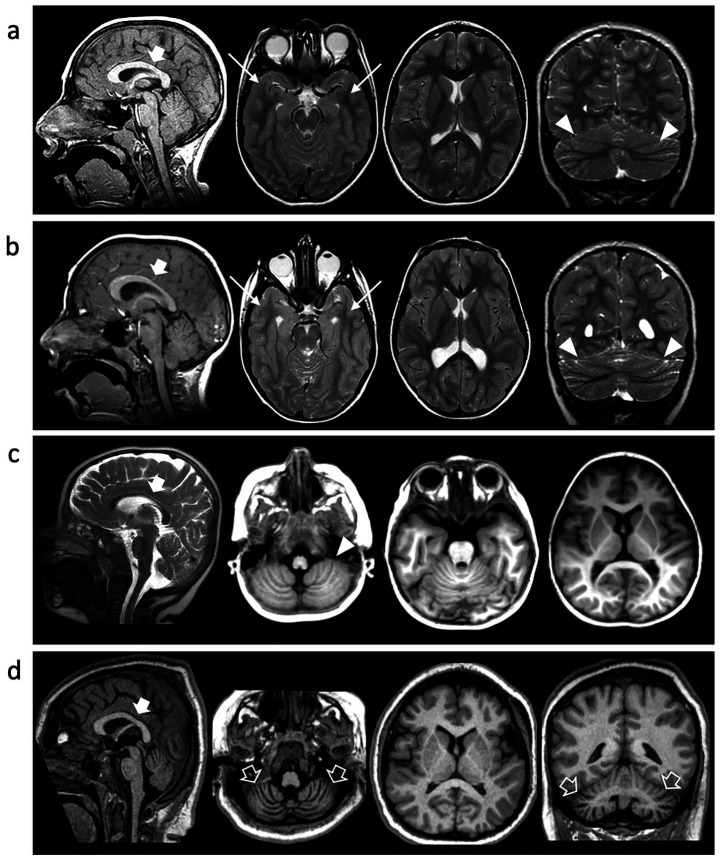
Neuroimaging findings. Brain MRI of Individual 1 at 10y **(A)**, Individual 2 at 7 y **(B)** and Individual 4 at 2 y **(C)** and 10y **(D)**. Sagittal T1 or T2-weighted images (first column), axial images (second and third columns), and coronal images (last column). Microcephaly with disproportionally large corpus callosum (thick arrows), simplified gyral pattern and cerebellar foliar anomalies are noted in all cases (arrowheads). There is bilateral temporo-basal dysgyria in Individual 1 and Individual 2 (arrows). Progressive cerebellar atrophy is detected in Individual 3 over the course of 8 years (empty arrows).

A 15-year-old boy, born at term following an uneventful pregnancy, exhibited postnatal microcephaly and turricephaly during early infancy. Early neurodevelopment was marked by global delay, reduced muscle tone, impaired primitive reflexes, and poor verbal output limited to non-purposeful syllables. By age 2 years, exaggerated deep tendon reflexes and ataxic gait emerged. Formal neuropsychiatric evaluation confirmed features consistent with ASD, including impaired communication and relational functioning. On his examination at our department at the age of 4 years, microcephaly was documented at -2SD and behavioral stereotypies, sensory-seeking behaviors, and tiptoe walking were also documented. ADOS-2 confirmed ASD diagnosis, and Risperidone was introduced with partial behavioral benefit. Follow-up at the age of 10 years revealed improved receptive language but persistent absence of expressive speech. Brain MRI at this age showed microcephaly with disproportionally large corpus callosum, simplified gyral pattern, bilateral temporo-basal dysgyria, and cerebellar foliar anomalies (Figure 3). Array-CGH identified a paternally inherited 16p13.11 duplication of uncertain clinical significance. WES revealed a novel *TUBB2A* variant (c.1172G > A; p.Arg391His). The variant was absent in the parents.

#### 3.4.2 Individual 2

The younger brother of Individual 1, currently 12 years old, also presented with global developmental delay and hypotonia. Motor milestones were severely delayed; unsupported walking began at 20 months. Language development was significantly impaired, with first intelligible words spoken at 4 years. By age 6, he exhibited hyperactivity, motor stereotypies, poor comprehension, and limited verbal output. Diagnosed with moderate ASD and ID, he required behavioral management with Risperidone.

At age 7, he developed sleep-related seizures characterized by myoclonus and tonic posturing. Seizure control was achieved with Clobazam after failure of Valproic Acid. At 9 years, EEG showed mild anterior-predominant abnormalities. Brain MRI revealed microcephaly with disproportionally large corpus callosum, simplified gyral pattern, temporo-basal dysgyria, thinning of the periventricular white matter with symmetric ventriculomegaly, and cerebellar foliar anomalies (Figure 3). Genetic testing was negative by array-CGH. WES confirmed the same heterozygous *TUBB2A* p.Arg391His variant identified in his brother. The variant was absent in the parents.

#### 3.4.3 Individual 3

Individual 3 is a 7-year-old female, born full-term with congenital hip dislocation, exhibited postnatal microcephaly (−1 SD at birth, progressing to −3.5 SD at 5 years). Gross motor milestones were profoundly delayed; independent walking was achieved at 6 years, although she primarily crawled or scooted. Examination revealed generalized hyperlaxity and increased reflexes without spasticity. Expressive language was absent, though receptive comprehension and social engagement (e.g., eye contact, smiling) were preserved. EEGs at 1 and 4 years were normal. MRI at age 1 revealed anterior-predominant simplified gyration without subcortical or white matter abnormalities. Array-CGH was normal. WES identified a *de novo TUBB2A* c.1172G > A (p.Arg391His) variant.

#### 3.4.4 Individual 4

Individual 4 is a 14-year-old female born to unrelated parents. The patient has microcephaly and global developmental delay with progressive loss of motor milestones; she initially was able to sit without support and stand with support, but by the age of 4 was no longer able to sit unsupported. The patient is currently non-verbal and with limited communication. She developed refractory epilepsy with multiple seizure types and subsequently manifested hyperkinetic movement disorders including choreoathetosis. Respiratory compromise emerged at the age of 10 years, necessitating tracheostomy and G-tube feeding due to neuromuscular dysphagia. She also developed progressive (neuromuscular) scoliosis and is wheelchair-dependent requiring adaptive equipment. Neuroimaging at age 2 showed microcephaly with disproportionally large corpus callosum, simplified gyral pattern, and cerebellar foliar anomalies, and by age 10, marked cerebellar atrophy and periatrial white matter signal changes were evident. WES identified the same *de novo TUBB2A* variant (c.1172G > A; p.Arg391His).

## 4 Discussion

The assembly of *α*- and *β*-tubulin isotypes into microtubules is a fundamental process underlying neuronal development and connectivity. These microtubule networks mediate diverse cellular events, including neurogenesis, neuronal migration, axonal guidance, and the formation of both cortical and subcortical brain architecture (Bahi-Buisson et al., 2014; Cushion et al., 2013; Breuss et al., 2012). Perturbations in tubulin dynamics are increasingly recognized as the molecular basis of a broad and clinically heterogeneous group of neurodevelopmental disorders termed tubulinopathies (Bahi-Buisson et al., 2014; Romaniello et al., 2018). These disorders arise from heterozygous pathogenic variants in genes encoding α- or β-tubulins, and are commonly associated with a spectrum of malformations of cortical development (MCD), as well as anomalies affecting subcortical structures such as the basal ganglia, corpus callosum, and cerebellum (Bahi-Buisson et al., 2014; Cushion et al., 2014; Cushion et al., 2013; Brock et al., 2021; Schmidt et al., 2021).

In this study, we describe four individuals carrying a novel recurrent *TUBB2A* missense variant (NM_001069.3:c.1172G > A; NP_001060.1:p.Arg391His), expanding the mutational and phenotypic spectrum associated with *TUBB2A*-related tubulinopathy. Importantly, the recurrence of the p.Arg391His variant across unrelated families raises the possibility of a mutational hotspot or, alternatively, low-level germline mosaicism, which may underlie its repeated occurrence.

Interestingly, all subjects presented with microcephaly and a simplified gyral pattern at the time of presentation. Temporo-basal dysgyria was observed in two individuals, and cerebellar foliar anomalies were noted in three cases. The corpus callosum appeared disproportionately large relative to the degree of microcephaly. Notably, none of the patients exhibited the hallmark features of tubulinopathy, such as asymmetric ventriculomegaly and basal ganglia dysmorphisms. Moreover, dysgyria was noted only in two cases and was limited to the temporo-basal regions. One individual demonstrated a progressive phenotype, developing cerebellar atrophy over an eight-year period. Cerebellar atrophy is, in fact, a recognized feature in certain tubulinopathies, particularly those linked to mutations in the *TUBB4A* gene, such as Hypomyelination with Atrophy of the Basal Ganglia and Cerebellum.

We systematically compared these individuals with the 24 previously reported cases in the literature, resulting in a combined cohort of 28 patients (Table 1) (Cushion et al., 2013; Brock et al., 2021; Schmidt et al., 2021; Cai et al., 2020; Sferra et al., 2018; Rodan et al., 2017; Lee et al., 2014). Analysis of this cohort reveals a highly penetrant core phenotype characterized by neurodevelopmental delay or intellectual disability in all individuals (100%), predominantly of moderate-to-severe severity (89.2%, 25/28). Language impairment was universally present (100%), with complete absence of speech in 64.2% (18/28). Autism spectrum disorder (ASD) or ASD traits were noted in 39.2% (11/28), reinforcing the neuropsychiatric vulnerability associated with *TUBB2A* dysfunction.

Postnatal microcephaly was present in nearly half of the patients (46.4%, 13/28), underscoring the role of *TUBB2A* in early neurogenesis and brain volume regulation. Epilepsy was a prominent comorbidity, affecting 78.5% (22/28), with 63.6% of cases manifesting seizures before 12 months of age. Seizure onset varied widely, ranging from infancy to mid-childhood. Motor deficits were reported in 82.1% (23/28), including axial hypotonia (35.7%), spastic diplegia or paraplegia (7.1%), and marked delays in achieving motor milestones (75%). Dysmorphic facial features were present in 28.5% (8/28), and visual impairment in 14.2% (4/28), though these features were not uniformly observed. Neuroimaging findings further support the pathogenic role of *TUBB2A* variants. MRI abnormalities were present in all individuals, with 67.8% (19/28) exhibiting cortical malformations. Pachygyria (21.4%) and diffuse dysgyria (32.1%) were the most common cortical anomalies, while dysgenesis or thinning of the corpus callosum was observed in 64.2% (18/28). Additional structural changes included cerebellar vermis or hemispheric hypoplasia (21.4%), basal ganglia dysplasia (21.4%), white matter abnormalities (25%), and ventricular enlargement (10.7%). These imaging findings emphasize the widespread disruption of both cortical and subcortical development resulting from *TUBB2A* dysfunction.

Among our newly described individuals, two (Individuals 1 and 2) are siblings born to unaffected, non-consanguineous parents. Sanger sequencing confirmed the absence of the variant in both parents, indicating germline mosaicism as the likely mechanism of transmission although additional tests were nor performed based on parents’ decision. This is the first documented case of *TUBB2A*-related tubulinopathy due to gonadal mosaicism, contrasting with all previously published *TUBB2A* variants, which have arisen *de novo*. This finding underscores the importance of considering recurrence risk in familial settings, even when initial testing suggests de novo origin. The identified variant, p.Arg391His, affects a highly conserved residue within the C-terminal domain of *β*-tubulin, localized at the longitudinal E-site heterodimer interface—a critical surface for *α*/β-tubulin dimerization (Figure 1E). To assess the structural and energetic consequences of this mutation, we employed high-resolution molecular modeling based on cryo-electron microscopy structures of human α/β-tubulin heterodimers (PDB IDs: 8ixa, 8ixd, 8ixf for TUBA1A-TUBB2A, TUBA1C-TUBB2A, and TUBA4A-TUBB2A, respectively). In all three models, Arg391 of β-tubulin forms conserved electrostatic and hydrophobic interactions with Tyr262, Trp346, and Glu434 of the α-tubulin subunit (Figures 2A–2D), stabilizing the heterodimer interface. The introduction of the Arg391His substitution was found to disrupt this conserved interaction network. The imidazole side chain of histidine is unable to replicate the native arginine’s interaction profile, resulting in altered side-chain packing and reduced electrostatic complementarity. Binding free energy calculations revealed a consistent energetic penalty across all three heterodimer models, with higher binding free energies in mutant complexes, indicating weakened dimerization (Figure 2E). Further analysis of the electrostatic surface potential revealed loss of electrostatic complementarity in all mutant complexes, with reduced Pearson correlation coefficients relative to wild-type pairs (Figure 2F). While solvation energies were slightly more favorable in mutant dimers (data not shown), this minor gain did not compensate for the loss of stabilizing electrostatic interactions. These findings strongly support a destabilizing effect of the Arg391His mutation on *α*/*β*-tubulin heterodimer formation, which may underlie the observed neurodevelopmental phenotype by compromising microtubule integrity and dynamics during early brain development (Soler et al., 2025).

Taken together, the convergence of clinical, neuroimaging, genetic, and computational data reinforces the role of Arg391 as a mutational hotspot in *TUBB2A*, with key structural and functional implications for microtubule assembly.

This study provides a comprehensive clinical and molecular characterization of *TUBB2A*-related neurodevelopmental disorders, incorporating four newly described patients—including the first familial recurrence due to gonadal mosaicism—and a detailed comparison with previously reported cases. The identification of a novel pathogenic variant, p.Arg391His, at a highly conserved and structurally critical interface, provides important insights into genotype–phenotype relationships and disease mechanisms. Through integrative structural modeling, we demonstrate that Arg391 plays a key role in maintaining heterodimer stability across multiple *α*-tubulin isoforms. Its substitution by histidine disrupts a conserved binding network, weakening α/*β*-tubulin dimerization and, by extension, microtubule function. These findings add to growing evidence that *TUBB2A* mutations can produce a spectrum of tubulinopathy phenotypes through impaired microtubule assembly.

However, our study has certain limitations. The lack of functional *in vivo* evidence—such as experiments using cultured cells or patient-derived neuronal models—restricts our ability to fully evaluate the physiological relevance of the p.Arg391His variant in microtubule dynamics. Notably, Luscan and colleagues conducted insightful *in vivo* studies on the same residue (c.1172G > A [p.Arg391His]; c.1171C > T [p.Arg391Cys]) in TUBB4B, employing COS-7 cell transfection and patient fibroblast assays. Their findings demonstrated deleterious effects of these substitutions on microtubule repolymerization dynamics and growth rates (Luscan et al., 2017). Given the high structural homology among tubulin subunit genes, these results provide valuable context for interpreting our observations.

Future analogous studies should aim to elucidate the impact of *TUBB2A* mutations in neuronal models and to investigate whether therapeutic stabilization of dimer interfaces might offer a viable intervention strategy. The recurrent involvement of Arg391 positions this site as a candidate target for future precision medicine approaches, with implications for both diagnosis and therapy in tubulinopathy-related neurodevelopmental disorders.

## Data Availability

The datasets presented in this study can be found in online repositories. The names of the repository/repositories and accession number(s) can be found below: https://zenodo.org/records/15869597, 15869597.

## Footnotes

^1^ https://www.ebi.ac.uk/jdispatcher/msa/clustalo

^2^ https://www.proteinatlas.org/ENSG00000137267-TUBB2A/brain

^3^ https://gnomad.broadinstitute.org
